# Supplementary material for: Cryo-EM structures demonstrate human IMPDH2 filament assembly tunes allosteric regulation
Source: eLife. 2020 Jan 30;9:e53243. doi: 10.7554/eLife.53243 (PMC7018514; doi:10.7554/eLife.53243)
Supplement: Figure 3—source data 1. [file elife-53243-fig3-data1.docx]

| Sample | IMPDH2 + ATP (0.5 mM), IMP (2 mM), NAD^+^ (2 mM) | | | |
| --- | --- | --- | --- | --- |
| Data collection and processing | # of micrographs | 2169 |  |  |
|  | Nominal magnification | 130,000 |  |  |
|  | Voltage (kV) | 300 |  |  |
|  | Electron fluence (e^-^/Å^2^) | 100 |  |  |
|  | Defocus range (μM) | 0.9 – 3.2 |  |  |
|  | Pixel size (Å) | 1.05 |  |  |
|  |  |  |  |  |
|  | Cryo-EM Reconstruction | Filament  assembly interface | Fully extended filament segment | Bent (1:3 C:E) filament segment |
|  | EMDB ID | EMD-20687 | EMD-20688 | EMD-20690 |
|  | Number of particles | 78471 | 9124 | 16819 |
|  | Symmetry imposed | D4 | D4 | D1 |
|  | Map resolution (Å) | 3.03 | 3.29 | 3.91 |
|  | FSC threshold | 0.143 | 0.143 | 0.143 |
|  | Map Resolution range (Å) | 2.8 – 3.6 | 3.0 – 5.5 | 3.6 – 10.5 |
|  |  |  |  |  |
| Refinement | PDB ID | 6U8E | 6U8N | 6U8R |
|  | Map sharpening | LocScale | LocScale | LocScale |
|  | Model composition |  |  |  |
|  | Non-hydrogen atoms | 22984 | 31528 | 31528 |
|  | Protein residues | 3000 | 4016 | 4016 |
|  | Ligands | 16 | 32 | 32 |
|  | Mean B factors (Å^2^) |  |  |  |
|  | Protein | 114.6 | 137.4 | 137.4 |
|  | Ligand | 123.1 | 162.1 | 173.3 |
|  | R.m.s. deviations |  |  |  |
|  | Bond lengths (Å) | 0.0111 | 0.0149 | 0.0138 |
|  | Bond angles (°) | 1.27 | 1.66 | 1.63 |
|  | Validation |  |  |  |
|  | MolProbity score | 1.88 | 2.67 | 2.43 |
|  | EMRinger score | 3.91 | 2.85 | 2.14 |
|  | Clashscore | 5.63 | 8.02 | 10.16 |
|  | Poor rotamers (%) | 2.02 | 9.05 | 3.21 |
|  | Ramachandran plot |  |  |  |
|  | Outliers (%) | 0 | 0.40 | 0.28 |
|  | Allowed (%) | 4.88 | 8.27 | 8.74 |
|  | Favored (%) | 95.12 | 91.33 | 90.98 |

**Figure 3—source data 1.** **Statistics of cryo-EM data collection, reconstruction and model refinement for the ATP/IMP/NAD^+^ dataset.**
