# Supplementary material for: Cryo-EM structures demonstrate human IMPDH2 filament assembly tunes allosteric regulation
Source: eLife. 2020 Jan 30;9:e53243. doi: 10.7554/eLife.53243 (PMC7018514; doi:10.7554/eLife.53243)
Supplement: Figure 3—source data 2. [file elife-53243-fig3-data2.docx]

| Sample | IMPDH2 + ATP (2 mM), NAD^+^ (2 mM) | | | |
| --- | --- | --- | --- | --- |
| Data collection and processing | # of micrographs | 2289 |  |  |
|  | Nominal magnification | 130,000 |  |  |
|  | Voltage (kV) | 300 |  |  |
|  | Electron fluence (e^-^/Å^2^) | 100 |  |  |
|  | Defocus range (μM) | 0.5 – 7.0 |  |  |
|  | Pixel size (Å) | 1.062 |  |  |
|  |  |  |  |  |
|  | Cryo-EM Reconstruction | Filament  assembly interface | Fully extended filament segment | Fully compressed filament segment |
|  | EMDB ID | EMD-20718 | EMD-20716 | EMD-20709 |
|  | Number of particles | 57979 | 13266 | 3394 |
|  | Symmetry imposed | D4 | D4 | D4 |
|  | Map resolution (Å) | 4.1 | 4.5 | 4.5 |
|  | FSC threshold | 0.143 | 0.143 | 0.143 |
|  | Map Resolution range (Å) | 3.8 – 7.8 | 4.3 – 7.0 | 4.2 – 7.3 |

**Figure 3—source data 2. Statistics of cryo-EM data collection, reconstruction and model refinement for the ATP/NAD^+^ dataset.**
