# Supplementary material for: Cryo-EM structures demonstrate human IMPDH2 filament assembly tunes allosteric regulation
Source: eLife. 2020 Jan 30;9:e53243. doi: 10.7554/eLife.53243 (PMC7018514; doi:10.7554/eLife.53243)
Supplement: Figure 3—source data 3. [file elife-53243-fig3-data3.docx]

| Sample | IMPDH2 + ATP (2 mM), IMP (3 mM) | | | |
| --- | --- | --- | --- | --- |
| Data collection and processing | # of micrographs | 2178 |  |  |
|  | Nominal magnification | 105,000 |  |  |
|  | Voltage (kV) | 300 |  |  |
|  | Electron fluence (e^-^/Å^2^) | 100 |  |  |
|  | Defocus range (μM) | 0.6 – 4.2 |  |  |
|  | Pixel size (Å) | 1.366 |  |  |
|  |  |  |  |  |
|  | Cryo-EM Reconstruction | Filament  assembly interface | Fully extended filament segment | Fully compressed filament segment |
|  | EMDB ID | EMD-20723 | EMD-20722 | EMD-20720 |
|  | Number of particles | 92587 | 9549 | 9648 |
|  | Symmetry imposed | D4 | D4 | D4 |
|  | Map resolution (Å) | 4.4 | 7.1 | 4.9 |
|  | FSC threshold | 0.143 | 0.143 | 0.143 |
|  | Map Resolution range (Å) | 4.2 – 6.7 | 5.5 – 8.1 | 4.2 – 10.5 |

**Figure 3—source data 3. Statistics of cryo-EM data collection, reconstruction and model refinement for the ATP/IMP dataset.**
