# Supplementary material for: Cryo-EM structures demonstrate human IMPDH2 filament assembly tunes allosteric regulation
Source: eLife. 2020 Jan 30;9:e53243. doi: 10.7554/eLife.53243 (PMC7018514; doi:10.7554/eLife.53243)
Supplement: Figure 4—source data 1. [file elife-53243-fig4-data1.docx]

| Sample | IMPDH2 + ATP (2 mM), GTP (2 mM) | |
| --- | --- | --- |
| Data collection and processing | # of micrographs | 1159 |
|  | Nominal magnification | 130,000 |
|  | Voltage (kV) | 300 |
|  | Electron fluence (e^-^/Å^2^) | 100 |
|  | Defocus range (μM) | 0.9 – 3.8 |
|  | Pixel size (Å) | 1.05 |
|  |  |  |
|  | Cryo-EM Reconstruction | Free canonical octamer |
|  | EMDB ID | EMD-20725 |
|  | Number of particles | 26847 |
|  | Symmetry imposed | D4 |
|  | Map resolution (Å) | 4.5 |
|  | FSC threshold | 0.143 |
|  | Map Resolution range (Å) | 4.1 – 5.7 |
|  |  |  |
| Refinement | PDB ID | 6UC2 |
|  | Map sharpening | LocScale |
|  | Model composition |  |
|  | Non-hydrogen atoms | 28408 |
|  | Protein residues | 3624 |
|  | Ligands | 24 |
|  | Mean B factors (Å^2^) |  |
|  | Protein | 243.6 |
|  | Ligand | 295.9 |
|  | R.m.s. deviations |  |
|  | Bond lengths (Å) | 0.0149 |
|  | Bond angles (°) | 1.93 |
|  | Validation |  |
|  | MolProbity score | 2.96 |
|  | EMRinger score | -0.62 |
|  | Clashscore | 12.18 |
|  | Poor rotamers (%) | 14.82 |
|  | Ramachandran plot |  |
|  | Outliers (%) | 0 |
|  | Allowed (%) | 7.61 |
|  | Favored (%) | 92.39 |

**Figure 4—source data 1. Statistics of cryo-EM data collection, reconstruction and model refinement for the ATP, 2 mM GTP dataset.**
