# Supplementary material for: Cryo-EM structures demonstrate human IMPDH2 filament assembly tunes allosteric regulation
Source: eLife. 2020 Jan 30;9:e53243. doi: 10.7554/eLife.53243 (PMC7018514; doi:10.7554/eLife.53243)
Supplement: Figure 4—source data 2. [file elife-53243-fig4-data2.docx]

| Sample | IMPDH2 + ATP (0.5 mM), GTP (20 mM), IMP (1 mM) | | | |
| --- | --- | --- | --- | --- |
| Data collection and processing | # of micrographs | 2353 |  |  |
|  | Nominal magnification | 165,000 |  |  |
|  | Voltage (kV) | 300 |  |  |
|  | Electron fluence (e^-^/Å^2^) | 40 |  |  |
|  | Defocus range (μM) | 0.3 – 2.8 |  |  |
|  | Pixel size (Å) | 0.827 |  |  |
|  |  |  |  |  |
|  | Cryo-EM Reconstruction | Filament assembly interface | Fully compressed filament segment | Fully compressed filament end |
|  | EMDB ID | EMD-20742 | EMD-20741 | EMD-20743 |
|  | Number of particles | 31246 | 8255 | 18063 |
|  | Symmetry imposed | D4 | D4 | C4 |
|  | Map resolution (Å) | 2.9 | 3.2 | 3.3 |
|  | FSC threshold | 0.143 | 0.143 | 0.143 |
|  | Map Resolution range (Å) | 2.7 – 3.5 | 3.0 – 5.9 | 3.0 – 6.0 |
|  |  |  |  |  |
| Refinement | PDB ID | 6UDP | 6UDO | 6UDQ |
|  | Map sharpening | LocScale | LocScale | LocScale |
|  | Model composition |  |  |  |
|  | Non-hydrogen atoms | 22728 | 31392 | 31008 |
|  | Protein residues | 3008 | 4008 | 3956 |
|  | Ligands | 8 | 32 | 32 |
|  | Mean B factors (Å^2^) |  |  |  |
|  | Protein | 68.5 | 87.0 | 71.1 |
|  | Ligand | 72.5 | 118.8 | 95.6 |
|  | R.m.s. deviations |  |  |  |
|  | Bond lengths (Å) | 0.0064 | 0.0080 | 0.0070 |
|  | Bond angles (°) | 1.21 | 1.32 | 1.27 |
|  | Validation |  |  |  |
|  | MolProbity score | 1.27 | 1.91 | 1.70 |
|  | EMRinger score | 5.25 | 3.34 | 3.74 |
|  | Clashscore | 3.95 | 4.93 | 5.04 |
|  | Poor rotamers (%) | 0.67 | 2.21 | 1.09 |
|  | Ramachandran plot |  |  |  |
|  | Outliers (%) | 0 | 0 | 0 |
|  | Allowed (%) | 2.43 | 5.86 | 6.00 |
|  | Favored (%) | 97.57 | 94.14 | 94.00 |

**Figure 4—source data 2. Statistics of cryo-EM data collection, reconstruction and model refinement for the ATP/IMP, 20 mM GTP dataset.**
