# Supplementary material for: Cryo-EM structures demonstrate human IMPDH2 filament assembly tunes allosteric regulation
Source: eLife. 2020 Jan 30;9:e53243. doi: 10.7554/eLife.53243 (PMC7018514; doi:10.7554/eLife.53243)
Supplement: Figure 5—source data 1. [file elife-53243-fig5-data1.docx]

| Sample | IMPDH2 + ATP (0.5 mM), GTP (2 mM), IMP (2 mM), NAD^+^ (2 mM) | | | | | | |
| --- | --- | --- | --- | --- | --- | --- | --- |
| Data collection and processing | # of micrographs | 2944 |  |  |  |  |  |
|  | Nominal magnification | 130,000 |  |  |  |  |  |
|  | Voltage (kV) | 300 |  |  |  |  |  |
|  | Electron fluence (e^-^/Å^2^) | 100 |  |  |  |  |  |
|  | Defocus range (μM) | 0.3 – 4.0 |  |  |  |  |  |
|  | Pixel size (Å) | 1.05 |  |  |  |  |  |
|  |  |  |  |  |  |  |  |
|  | Cryo-EM Reconstruction | Filament  assembly interface | Bent  (2:2 C:E) filament segment | Bent  (3:1 C:E) filament segment | Fully compressed filament segment | Free canonical octamer | Free interfacial octamer |
|  | EMDB ID | EMD-20691 | EMD-20704 | EMD-20705 | EMD-20701 | EMD-20707 | EMD-20706 |
|  | Number of particles | 166384 | 28918 | 49897 | 14067 | 17785 | 6770 |
|  | Symmetry imposed | D4 | D1 | D1 | D4 | D4 | D4 |
|  | Map resolution (Å) | 3.1 | 4.2 | 3.7 | 3.4 | 3.8 | 3.8 |
|  | FSC threshold | 0.143 | 0.143 | 0.143 | 0.143 | 0.143 | 0.143 |
|  | Map Resolution range (Å) | 3.0 – 3.8 | 3.9 – 8.9 | 3.3 – 8.7 | 3.1 – 5.1 | 3.6 – 5.4 | 3.4 – 6.8 |
|  |  |  |  |  |  |  |  |
| Refinement | PDB ID | 6U8S | 6UA2 | 6UA4 | 6U9O | 6UAJ | 6UA5 |
|  | Map sharpening | LocScale | LocScale | LocScale | LocScale | LocScale | LocScale |
|  | Model composition |  |  |  |  |  |  |
|  | Non-hydrogen atoms | 23080 | 31660 | 31726 | 31792 | 31032 | 23496 |
|  | Protein residues | 3008 | 4016 | 4016 | 4016 | 3952 | 3008 |
|  | Ligands | 16 | 36 | 38 | 40 | 40 | 16 |
|  | Mean B factors (Å^2^) |  |  |  |  |  |  |
|  | Protein | 149.3 | 218.6 | 134.3 | 142.6 | 155.6 | 98.1 |
|  | Ligand | 166.9 | 277.7 | 173.3 | 170.1 | 184.2 | 107.4 |
|  | R.m.s. deviations |  |  |  |  |  |  |
|  | Bond lengths (Å) | 0.0078 | 0.0129 | 0.0133 | 0.0141 | 0.0145 | 0.0104 |
|  | Bond angles (°) | 1.38 | 1.60 | 1.45 | 1.57 | 1.75 | 1.54 |
|  | Validation |  |  |  |  |  |  |
|  | MolProbity score | 2.59 | 2.71 | 2.18 | 2.56 | 2.97 | 2.53 |
|  | EMRinger score | 2.61 | 0.88 | 2.42 | 2.41 | 1.64 | 3.37 |
|  | Clashscore | 9.62 | 14.17 | 7.07 | 7.29 | 11.89 | 9.01 |
|  | Poor rotamers (%) | 9.06 | 4.10 | 2.60 | 8.31 | 12.03 | 8.52 |
|  | Ramachandran plot |  |  |  |  |  |  |
|  | Outliers (%) | 0 | 0.05 | 0.03 | 0 | 0 | 0 |
|  | Allowed (%) | 5.14 | 11.27 | 7.79 | 7.26 | 10.57 | 4.86 |
|  | Favored (%) | 94.86 | 88.68 | 92.19 | 92.74 | 89.43 | 95.14 |

**Figure 5—source data 1.** **Statistics of cryo-EM data collection, reconstruction and model refinement for the ATP/IMP/NAD^+^, 2 mM GTP dataset.**
