# Supplementary material for: Cryo-EM structures demonstrate human IMPDH2 filament assembly tunes allosteric regulation
Source: eLife. 2020 Jan 30;9:e53243. doi: 10.7554/eLife.53243 (PMC7018514; doi:10.7554/eLife.53243)
Supplement: Transparent reporting form [file elife-53243-transrepform.docx]

***eLife’s* transparent reporting form**

We encourage authors to provide detailed information *within their submission* to facilitate the interpretation and replication of experiments. Authors can upload supporting documentation to indicate the use of appropriate reporting guidelines for health-related research (see [EQUATOR Network](http://www.equator-network.org/%20)), life science research (see the [BioSharing Information Resource](https://biosharing.org/)), or the [ARRIVE guidelines](http://www.plosbiology.org/article/info:doi/10.1371/journal.pbio.1000412) for reporting work involving animal research. Where applicable, authors should refer to any relevant reporting standards documents in this form.

If you have any questions, please consult our Journal Policies and/or contact us: [editorial@elifesciences.org](mailto:editorial@elifesciences.org).

**Sample-size estimation**

- You should state whether an appropriate sample size was computed when the study was being designed
- You should state the statistical method of sample size computation and any required assumptions
- If no explicit power analysis was used, you should describe how you decided what sample (replicate) size (number) to use

Please outline where this information can be found within the submission (e.g., sections or figure legends), or explain why this information doesn’t apply to your submission:

The number of electron micrographs collected and the number of segments/particles extracted and processed during image analysis are summarized in Figure 2—figure supplement 1, Figure 3—figure supplement 1, Figure 3—source data 1, Figure 3—source data 2, Figure 3—source data 3, Figure 4—figure supplement 2, Figure 4—figure supplement 3, Figure 4—source data 1, Figure 4—source data 2, Figure 5—figure supplement 1, Figure 5—figure supplement 2, Figure 5—figure supplement 3, Figure 5—figure supplement 4, and Figure 5—source data 1.

Routine enzymatic assays were performed as technical replicates (performed on different days, with the same material), as described in Materials & Methods, subsection “IMPDH activity assays”

**Replicates**

- You should report how often each experiment was performed
- You should include a definition of biological versus technical replication
- The data obtained should be provided and sufficient information should be provided to indicate the number of independent biological and/or technical replicates
- If you encountered any outliers, you should describe how these were handled
- Criteria for exclusion/inclusion of data should be clearly stated
- High-throughput sequence data should be uploaded before submission, with a private link for reviewers provided (these are available from both GEO and ArrayExpress)

Please outline where this information can be found within the submission (e.g., sections or figure legends), or explain why this information doesn’t apply to your submission:

Cryo-EM maps were determined once. Routine enzyme activity assays (Figure 2, Figure 5, and Figure 5—figure supplement 1) were performed in triplicate as described in Methods.

**Statistical reporting**

- Statistical analysis methods should be described and justified
- Raw data should be presented in figures whenever informative to do so (typically when N per group is less than 10)
- For each experiment, you should identify the statistical tests used, exact values of N, definitions of center, methods of multiple test correction, and dispersion and precision measures (e.g., mean, median, SD, SEM, confidence intervals; and, for the major substantive results, a measure of effect size (e.g., Pearson's r, Cohen's d)
- Report exact p-values wherever possible alongside the summary statistics and 95% confidence intervals. These should be reported for all key questions and not only when the p-value is less than 0.05.

Please outline where this information can be found within the submission (e.g., sections or figure legends), or explain why this information doesn’t apply to your submission:

Experimental cryo-EM maps were evaluated by Fourier shell correlation between independently refined halves of data, and local resolution assessement, as described in Materials & Methods, and Figure 2—figure supplement 1, Figure 3—figure supplement 1, Figure 4—figure supplement 2, Figure 4—figure supplement 3, and Figure 5—figure supplement 1, Figure 5—figure supplement 2, Figure 5—figure supplement 3, Figure 5—figure supplement 4. Refinement and Validation statistics, including MolProbity, Ramachandran, and EMRinger scores, are summarized in Figure 3—source data 1, Figure 3—source data 2, Figure 3—source data 3, Figure 4—source data 1, Figure 4—source data 2, and Figure 5—source data 1.

Enzyme activities (Figure 2, Figure 5, and Figure 5—figure supplement 1) are the average of triplicate measurements with error bars indicating standard deviation as described in the figure legends.

(For large datasets, or papers with a very large number of statistical tests, you may upload a single table file with tests, Ns, etc., with reference to sections in the manuscript.)

**Group allocation**

- Indicate how samples were allocated into experimental groups (in the case of clinical studies, please specify allocation to treatment method); if randomization was used, please also state if restricted randomization was applied
- Indicate if masking was used during group allocation, data collection and/or data analysis

Please outline where this information can be found within the submission (e.g., sections or figure legends), or explain why this information doesn’t apply to your submission:

Structures were determined once and enzyme assays were in triplicate, allocation into groups does not apply.

**Additional data files (“source data”)**

- We encourage you to upload relevant additional data files, such as numerical data that are represented as a graph in a figure, or as a summary table
- Where provided, these should be in the most useful format, and they can be uploaded as “Source data” files linked to a main figure or table
- Include model definition files including the full list of parameters used
- Include code used for data analysis (e.g., R, MatLab)
- Avoid stating that data files are “available upon request”

Please indicate the figures or tables for which source data files have been provided:

All source data tables (Figure 3—source data 1, Figure 3—source data 2, Figure 3—source data 3, Figure 4—source data 1, Figure 4—source data 2, and Figure 5—source data 1) are summaries of cryo-EM data collection, processing, refinement, and validation. The atomic coordinates into the PDB under accession codes 6U8E, 6U8N, 6U8R, 6U8S, 6U9O, 6UA2, 6UA4, 6UA5, 6UAJ, 6UC2, 6UDP, 6UDO, and 6UDQ. The cryo-EM maps have been deposited into the EMDB under accession codes 20687, 20688, 20690, 20691, 20701, 20704, 20705, 20706, 20707, 20709, 20716, 20718, 20720, 20722, 20723, 20725, 20742, 20741, and 20743.
